# Bioinformatics analysis to identify the relationship between human papillomavirus-associated cervical cancer, toll-like receptors and exomes: A genetic epidemiology study

**DOI:** 10.1371/journal.pone.0305760

**Published:** 2024-08-29

**Authors:** Fabiana de Campos Gomes, Deizyane dos Reis Galhardo, Aline Carvalho Gonçalves Navegante, Gabriela Sepêda dos Santos, Helana Augusta Andrade Leal Dias, José Ribamar Leal Dias Júnior, Marie Esther Pierre, Marlucia Oliveira Luz, João Simão de Melo Neto

**Affiliations:** 1 Postgraduate Program in Collective Health in the Amazon (PPGSCA), Federal University of Pará (UFPA), Belém, Pará, Brazil; 2 Faculty of Medicine CERES (FACERES), São José do Rio Preto, São Paulo, Brazil; National Institute of Biologicals (NIB), Ministry of Health & Family Welfare, Government of India, INDIA

## Abstract

**Introduction:**

Genetic variants may influence Toll-like receptor (TLR) signaling in the immune response to human papillomavirus (HPV) infection and lead to cervical cancer. In this study, we investigated the pattern of TLR expression in the transcriptome of HPV-positive and HPV-negative cervical cancer samples and looked for variants potentially related to TLR gene alterations in exomes from different populations.

**Materials and methods:**

A cervical tissue sample from 28 women, which was obtained from the Gene Expression Omnibus database, was used to examine TLR gene expression. Subsequently, the transcripts related to the TLRs that showed significant gene expression were queried in the Genome Aggregation Database to search for variants in more than 5,728 exomes from different ethnicities.

**Results:**

Cancer and HPV were found to be associated (p<0.0001). *TLR1*(p = 0.001), *TLR3*(p = 0.004), *TLR4*(221060_s_at)(p = 0.001), TLR7(p = 0.001;p = 0.047), *TLR8*(p = 0.002) and *TLR10*(p = 0.008) were negatively regulated, while *TLR4*(1552798_at)(p<0.0001) and *TLR6*(p = 0.019) were positively regulated in HPV-positive patients (p<0.05). The clinical significance of the variants was statistically significant for *TLR1*, *TLR3*, *TLR6* and *TLR8* in association with ethnicity. Genetic variants in different TLRs have been found in various ethnic populations. Variants of the TLR gene were of the following types: *TLR1*(*5_prime_UTR*), *TLR4*(*start_lost*), *TLR8*(*synonymous;missense*) and *TLR10*(*3_prime_UTR*). The “*missense*” variant was found to have a risk of its clinical significance being pathogenic in South Asian populations (OR = 56,820[95%CI:40,206,80,299]).

**Conclusion:**

The results of this study suggest that the variants found in the transcriptomes of different populations may lead to impairment of the functional aspect of TLRs that show significant gene expression in cervical cancer samples caused by HPV.

## Introduction

Cervical cancer is the fourth most common gynecologic cancer in women, with more than 600,000 new cases and more than 340,000 deaths worldwide in 2020. In Brazil, it is the third most common neoplasm in women [1, 2]. It can originate in the endometrium or myometrium [3] and manifests itself through intralesional cervical lesions caused mainly by human papillomavirus (HPV), which alters the host genome [4]. Long-term exposure to high-risk HPV is an important risk factor for cervical intraepithelial lesions [5].

Globally, HPV infection by women’s income in 2018 was 71.7% for low-income women, 72.8% for middle-income women, and 45.7% for high-income women. These high rates may be related to the incidence of cervical cancer in poorer countries [6]. In relation to the age group, younger women are more prone to HPV infection; however, the most serious diagnoses, both in cytology and in biopsies related to cancer, prevail in older women [7].

HPV is a small double-stranded DNA virus, and its oncogenic properties are due to the E6 and E7 oncoproteins, which act by inhibiting the tumor suppressors p53 and pRB [8]. Cervical cancer can be caused by different HPV types, which are classified into high- and low-risk groups. The high-risk groups include the following types: 16, 18, 31, 33, 35, 39, 45, 51, 52, 56, 58, 59, and 68, while the low-risk group includes the following types: 6, 11, 40, 42, 43, 44, 54, 61, 70, 72, and 81. In addition to the type of HPV that leads to susceptibility to cervical cancer, the tumor microenvironment may influence the expression of some Toll-like receptors (TLRs), which are closely related to HPV infection and cervical cancer. In a study conducted in the United States [9], *TLR2* and *TLR4* expression levels were found to be greater in cervical cancer and premalignant lesions than in normal controls [10].

Previous studies have shown the association between the expression of TLRs and the clinicopathological features of cervical cancer patients [11]. TLRs play an important role in the natural defense against HPV infection [5, 12–14]. TLRs recognize exogenous and endogenous pathogens that can be associated with the innate or acquired immune response [15, 16]. Currently, there are ten different TLRs, described as follows: *TRL1*, *TLR2*, *TLR3*, *TLR4*, *TLR5*, *TLR6*, *TLR7*, *TLR8*, *TLR9*, and *TLR10*. They can be expressed at different sites in the cell. *TLR1*, *TLR2*, *TLR4*, *TLR5*, *TLR6*, and *TLR10* are located at the plasma membrane and recognize the lipid structure of the surface of the pathogenic biological agent, while *TLR3*, *TLR7*, *TLR8*, and *TLR9* are found in the cell cytoplasm and identify the nucleic acid arrangement of the pathogenic organism [10, 15]. Among the diversity of receptors, *TLR10* is the most recently described, generating controversy regarding its function; *TLR4* recognizes exogenous pathogens and is closely linked to the growth of HPV-positive cervical cancer [17].

HPV virus can affect the expression of TLRs receptors and control their signaling pathways, promoting persistent infection, a factor that can lead to cervical cancer due to the cervical lesions caused [10]. There is evidence in the literature that factors other than HPV infection, including altered cytokine expression, differential TLR expression, and genetic variants found in TLRs, contribute to the development of cervical cancer [18]. A recent study analyzed exomes from individuals with HPV-associated cervical cancer and revealed *missense* variants in the genes [19]. In addition, genetic variation in TLR genes associated with inflammation has been associated with susceptibility to cancer formation and pathogenic infections [20].

In this study, we investigated the pattern of TLR expression in the transcriptome of HPV-positive and HPV-negative cervical cancer samples and screened for variants possibly related to TLR gene modifications in exomes from different populations.

## Materials and methods

### 1. Ethical aspects

The Gene Expression Omnibus (GEO) and Genome Aggregation Database (gnomAD) databases are publicly available and provide researchers with guidelines and regulations to ensure the confidentiality of data made available on the platform in accordance with the principles of ethics in research with human subjects. Since the data are publicly available, the National Health Council of Brazil, in accordance with Resolution No. 466 of 2012 and Supplementary No. 510 of 07 April 2016, waives the assessment by the Ethics Committee for Research Involving Human Subjects.

### 2. Study design

This was an ecological, descriptive and inferential observational study.

### 3. Database GEO

To examine gene expression in HPV-positive and HPV-negative uterine cancer epithelial samples, data were extracted from the GEO platform functional genomics data repository, which is publicly available at the National Center for Biotechnology Information (NCBI) (https://www.ncbi.nlm.nih.gov/gds/ accessed July 21, 2023).

For the search, the words “HPV positive” with Boolean operator “and” “HPV negative” and Boolean operator “and” “cervical cancer” were used. The details of the survey were “((HPV[All Fields] AND positive[All Fields]) AND (HPV[All Fields] AND negative[All Fields]) AND (“uterine cervical neoplasms”[MeSH Terms] OR cervical cancer[All Fields])) AND “*Homo sapiens*”[porgn] AND “Expression profiling by array”[Filter]”.

Results with positive and negative HPV results and information on age, histological grade, cancer stage and metastasis status were selected. Thus, the GSE6791 series was deposited by [21] from the Department of Oncology at the University of Wisconsin-Madison, USA.

#### 3.1. Inclusion and exclusion criteria

The GSE6791 series contained samples of cervical cancer and head and neck cancer. In this study, we selected only normal cervical and cervical cancer samples from women of all ages who were HPV positive or HPV negative. All data from head and neck cancer samples were excluded from the analyses.

#### 3.2. Population and processing data

In this study, tissue samples were collected from the cervix of 28 women aged 25 to 66 years. The 28 cervical cancer samples collected corresponded to 17 HPV-positive cervical cancer samples (HPV-positive group), 3 HPV-negative cervical cancer samples (HPV-negative group), and 8 normal cervical tissue samples (control group). All tumor samples were collected before the patients were treated. Total human gene expression was verified using Affymetrix Human Genome U133 Plus 2.0 arrays [21].

Microarray data of cervical cancer samples and their respective controls were selected and downloaded from the Geo repository. TLR gene expression in cervical cancer tissue samples was determined based on the identification of the GSE6791 series probes, and the results are presented in a gene table generated by GEO2R (https://www.ncbi.nlm.nih.gov/geo/geo2r/?acc=GSE6791 accessed July 22, 2023).

After determining the gene expression of different TLRs in cervical tissue samples, the TLR probes of statistical significance found in this sample were subjected to exome sequencing investigation; these probes were considered probes of interest for this study. Thus, the transcriptome data related to *TLR1* (210176_at), *TLR3* (242667_at), *TLR4* (221060_s_at; 1552798_a_at), *TLR6* (207446_at), *TLR7* (222952_s_at; 220146_at), *TLR8* (220832_at) and *TLR10* (223750_s_at).

The respective probes of the identified TLRs were obtained from the Ensembl platform (https://grch37.ensembl.org/index.html accessed August 01, 2023), and protein-coding transcriptomes and non-protein-coding transcriptomes related to the probes of interest were collected. The following transcripts were detected: *TLR1* [(210176_at): ENST00000308979.2; ENST00000502213.2]; *TLR3* [(242667_at): ENST00000296795.3]; *TLR4* [(221060_s_at): ENST00000355622.6; ENST00000394487.4; ENST00000472304.1]; *TLR4* [(1552798_a_at): ENST00000355622.6; ENST00000394487.4; ENST00000472304.1]; *TLR6* [(207446_at): ENST00000381950.1; ENST00000436693.2]; *TLR7* [(222952_s_at):ENST00000380659.3]; *TLR7*[(220146_at): ENST00000380659.3], *TLR8* [(220832_at): ENST00000218032.6]; and *TLR10* [(223750_s_at): ENST00000502321.1].

### 4. Database gnomAD

To obtain access to exome sequencing data from different populations, the gnomAD platform was used (https://gnomad.broadinstitute.org accessed July 25, 2023).

#### 4.1. Inclusion and exclusion criteria

For the gnomAD data, 60,106 exome samples from different populations deposited in ExAC v1.0 were considered. However, only the exomes with the transcripts of interest were included in the analysis.

To identify the potential of variants in TLR, clinical significance was divided into the following categories: benign (benign, benign/likely benign and likely benign), pathogenic (pathogenic and conflicting interpretations of pathogenicity) and risk factor (likely risk allele and risk factor). Variants with uncertain clinical significance were excluded.

#### 4.2. Population and processing data

On the gnomAD platform, spreadsheets were downloaded with the data of the transcriptomes found. The transcriptomes identified belonged to the ethnicities “African and African American”, “Latino Admixed American”, “European Finnish”, “European Non-Finnish”, “East Asian” and “South Asian”. Of the 60,106 exome samples, only 5,728 belonged to the transcriptomics of interest.

Variants were screened using the transcripts of interest. Thus, the following variants were found in different populations: “*3_prime_UTR*”, “*5_prime_UT*R”, “*frameshift*”, “*inframe_deletion*”, “*intron*”, “*missense*”, “*splice_donor*”, “*splice_region*”, “*start_lost*”, “*stop_gained”* e “*synonymous*”, in different populations. The effect of genetic variation on protein expression was evaluated via the Ensembl platform (https://www.ensembl.org/info/genome/variation/prediction/predicted_data.html accessed August 25, 2023).

### 5. Identification of pathogenic variants

The variants classified as pathogenic or probable pathogenic were considered to identify the risk of the variants causing mutations in alleles of different populations from the transcripts that were significant when associating the variants with ethnicity. In the sample of 5,728 exomes, only variants of the “*missense*” type were found to have pathogenic and likely pathogenic potential.

**Fig 1:**
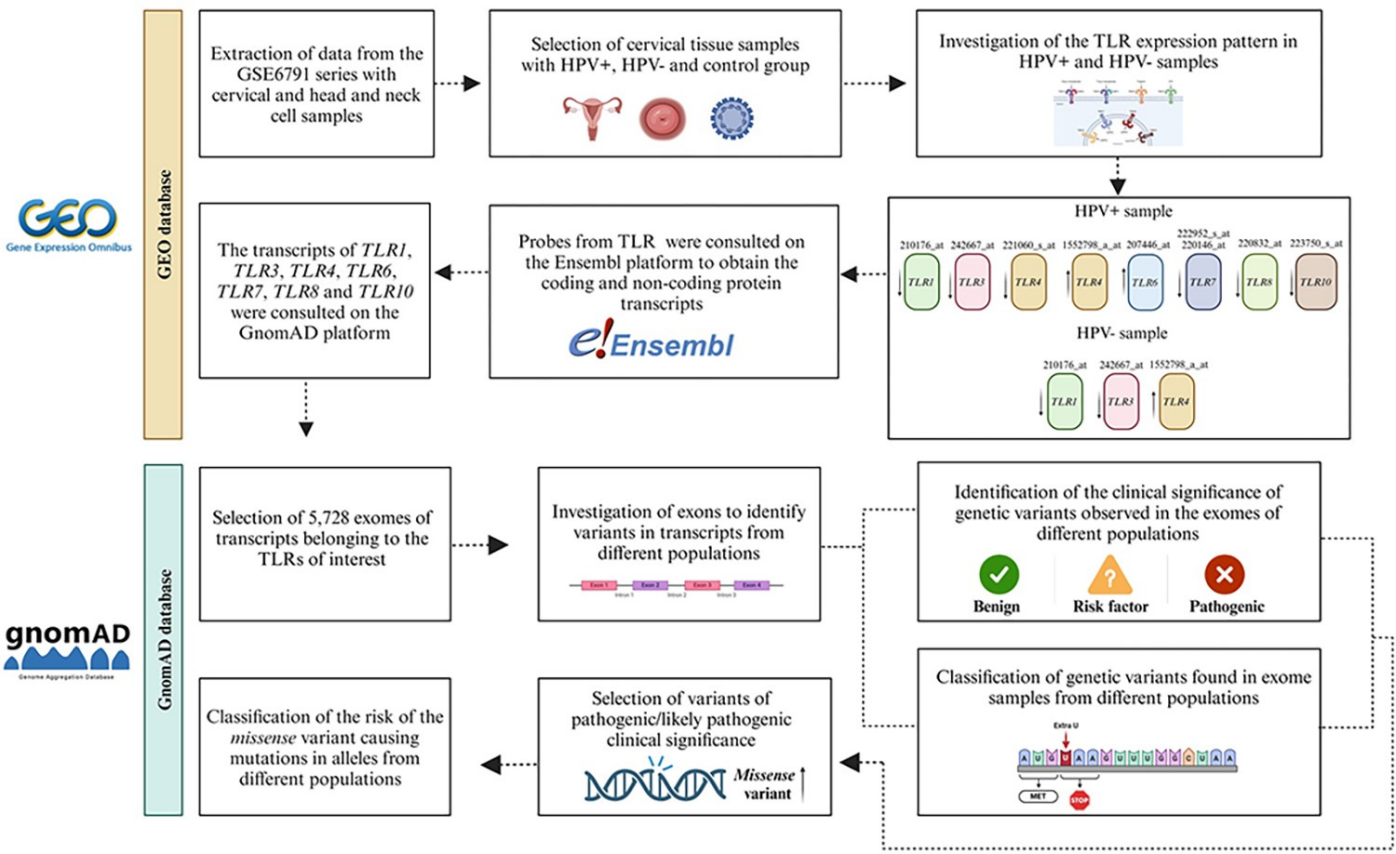
Graphical pipeline with the main points of the methods. The figure illustrates the methodological workflow, highlighting the key stages.

Fig 1 shows a graphical pipeline describing the main steps of the methodology used in this study.

### 6. Statistical analyses

The descriptive data are presented as the absolute and relative frequencies, means and standard deviations (parametric data) or medians and ranges (nonparametric data). To verify normality, the data were subjected to the Kolmogorov‒Smirnov test. To determine if there was a statistically significant difference between the variables, the chi-square test (χ^2^) was used, and a critical Z was adopted as a posttest for the associations from the χ^2^ test. The unpaired t test (t) was applied for nonparametric variables, and one-way analysis of variance (ANOVA) or the Kruskal‒Wallis test was used for parametric or nonparametric variables, respectively. The Bonferroni correction test was considered post hoc (F).

Gene expression analysis was performed using the false discovery rate (FDR) procedure by Benjamini & Hochberg. The log fold change threshold (LogFC) was considered to be 2. The odds ratio (OR) was calculated with 95% confidence intervals (95% CIs) and was used to quantify the risks of mutation in ethnic groups. Statistical significance was set at p < 0.05.

For statistical analyses, the Statistical Package for the Social Sciences (SPSS) version 25.0 was used (IBM Corp. Released 2017. IBM SPSS Statistics for Windows, version 25.0. IBM Corp., Armonk, NY, USA).

## Results

### 1. Analysis of cervical cancer associated with HPV

Initially, HPV-associated cervical cancer (χ^2^ = 17.309; p<0.0001) was tested and identified according to the base GEO (Table 1). As observed in Table 1, it is important to highlight that the sample was homogeneous regarding age, histological grade, stage and metastasis.

**Table 1:** Frequency of cervical cancer, age, histological grade, stage and metastasis in women with or without HPV.

Variables	HPV- (n = 11)	HPV+ (n = 17)	χ^2^ or t	p
**Age (years)**_Mean(σ)_	43.64(11.16)	44.06(10.164)	-0.103	0.918
**Tissue**_(%)_			17.309	<0.0001*
Cervical Cancer	10.7	60.7		
Cervical Normal	28.5	0		
**Histological grade**_(%)_			0.065	0.798
Poorly/undiffer	10	50		
Well/moderately	5	35		
**Stage**_(%)_			2.571	0.860
IB	5	40		
IB1	0	5		
IB2	10	20		
II	0	5		
II/III	0	5		
IIIB	0	5		
IV	0	5		
**Metastasis**_(%)_	5	15	0.392	0.531

χ^2^: chi-square test; t: unpaired t test; σ: standard deviation

*p < 0.05

### 2. Differential gene expression patterns of Toll-like receptors in cervical tissue samples

Table 2 presents the associations of cervical cancer with HPV and the expression of different TLR genes (*TLR1*, *TLR2*, *TLR3*, *TLR4*, *TLR5*, *TLR6*, *TLR7*, *TLR8*, *TLR8-AS1*, *TLR9* and *TLR10*). *TLR1* and *TLR3* (Id:242667_at) exhibited reduced expression, and *TLR4* (Id1552798_a_at) exhibited increased expression in individuals with cancer, independent of HPV (p < 0.05). However, the Log2 Fold-Change expression differential (LogFC) was greater in individuals who were HPV-positive for *TLR1* and *TLR3*. The LogFC was also greater for *TLR4*.

**Table 2:** Differential gene expression of Toll-like receptors (TLRs) in human papillomavirus-associated cervical cancer.

TLR	Id	Control (n = 8)	HPV- (n = 3)	HPV+ (n = 17)	FC (control *vs* HPV-)	FC (control *vs* HPV+)	LogFC (control *vs* HPV-)	LogFC (control *vs* HPV+)	F	*p*
*TLR1*	210176_at^&^	7.23 (0.87)a	6.45 (0.25)b	6.33 (1.43)b	0.89	0.87	-0.168	-0.200	14.20	0.001*
*TLR2*	204924_at^#^	7.10 (0.44)	6.74 (0.18)	6.68 (0.37)	-	-	-	-	3.25	0.056
*TLR3*	242667_at^&^	6.86 (0.98)a	6.46 (0.19)b	6.36 (0.88)b	0.94	0.93	-0.089	-0.104	11.22	0.004*
	206271_at^#^	8.64 (0.61)	8.12 (1.04)	8.94 (0.80)	-	-	-	-	1.59	0.224
	239587_at^#^	6.98 (0.23)	6.86 (0.17)	6.97 (0.29)	-	-	-	-	0.23	0.794
*TLR4*	221060_s_at^#^	6.74 (0.21)a	6.38 (0.17)	6.36 (0.21)b	-	0.94	-	-0.089	8.92	0.001*
	232068_s_at^#^	6.79 (0.45)	6.82 (1.57)	7.12 (1.17)	-	-	-	-	0.35	0.705
	224341_x_at^&^	4.07 (0.61)	4.06 (0.21)	4.18 (1.45)	-	-	-	-	3.23	0.199
	1552798_a_at^#^	7.88 (0.34)a	9.32 (0.80)b	9.32 (0.79)b	1.18	1.18	0.238	0.238	12.25	<0.0001*
*TLR5*	210166_at^#^	5.03 (0.15)	4.93 (0.22)	5.45 (1.07)	-	-	-	-	0.293	0.410
*TLR6*	207446_at^&^	5.22 (0.79)a	5.01 (1.91)	6.03 (3.85)b	-	1.15	-	0.201	7.95	0.019*
	239021_at^#^	4.74 (0.14)	4.69 (0.33)	4.97 (0.35)	-	-	-	-	2.15	0.137
*TLR7*	222952_s_at^#^	7.63 (0.51)a	7.01 (0.89)	6.86 (0.41)b	-	0.89	-	-0.168	8.87	0.001*
	220146_at^#^	7.08 (0.25)a	6.74 (0.10)	6.67 (0.42)b	-	0.94	-	-0.089	3.462	0.047*
*TLR8*	220832_at^#^	7.08 (0.19)a	6.70 (0.14)	6.53 (0.36)b	-	0.92	-	-0.120	8.42	0.002*
	229560_at^&^	6.32 (0.34)	6.18 (0.41)	6.16 (1.31)	-	-	-	-	3.56	0.168
*TLR8-AS1*	1562805_at^#^	6.15 (0.49)	6.59 (0.71)	6.71 (1.14)	-	-	-	-	0.924	0.410
*TLR9*	223903_at^&^	6.08 (1.62)	6.23 (0.46)	6.02 (4.03)	-	-	-	-	0.15	0.929
*TLR10*	223751_x_at^&^	4.58 (1.25)	4.52 (0.29)	4.79 (2.09)	-	-	-	-	4.71	0.095
	223750_s_at^#^	7.18 (0.30)a	6.93 (0.19)	6.81 (0.24)b	-	0.94	-	-0.089	5.84	0.008*

Id: probe identification; a, b: Bonferroni posttest; FC: fold change; LogFC: log2-fold change of differential expression; F: effect size

*p < 0.05; #: one-way ANOVA; &: Kruskal‒Wallis test.

The expression of *TLR4* (Id: 221060_s_at), *TLR7*, *TLR8* (Id220832_at) and *TLR10* (Id: 223750_s_at) was reduced in HPV-positive patients with negative LogFC values. In contrast, *TLR6* showed increased gene expression and LogFC in HPV-positive individuals. *TLR2*, *TLR5*, *TLR8-AS1* and *TLR9* showed no significant differences.

### 3. Clinical aspects of variants found in transcripts of exomes from different populations

After the identification of gene expression in the different TLRs in cervical cancer samples, the transcripts of the receptors that had significant gene expression in the samples were subjected to analysis of the clinical significance of the variants in the exomes of different populations.

The levels of the TLR4, TLR7 and TLR10 transcripts did not significantly differ among the populations (p > 0.05) when the associations between the clinical significance of the variants and ethnicity were analyzed (χ^2^ e Z_crit_).

**Fig 2:**
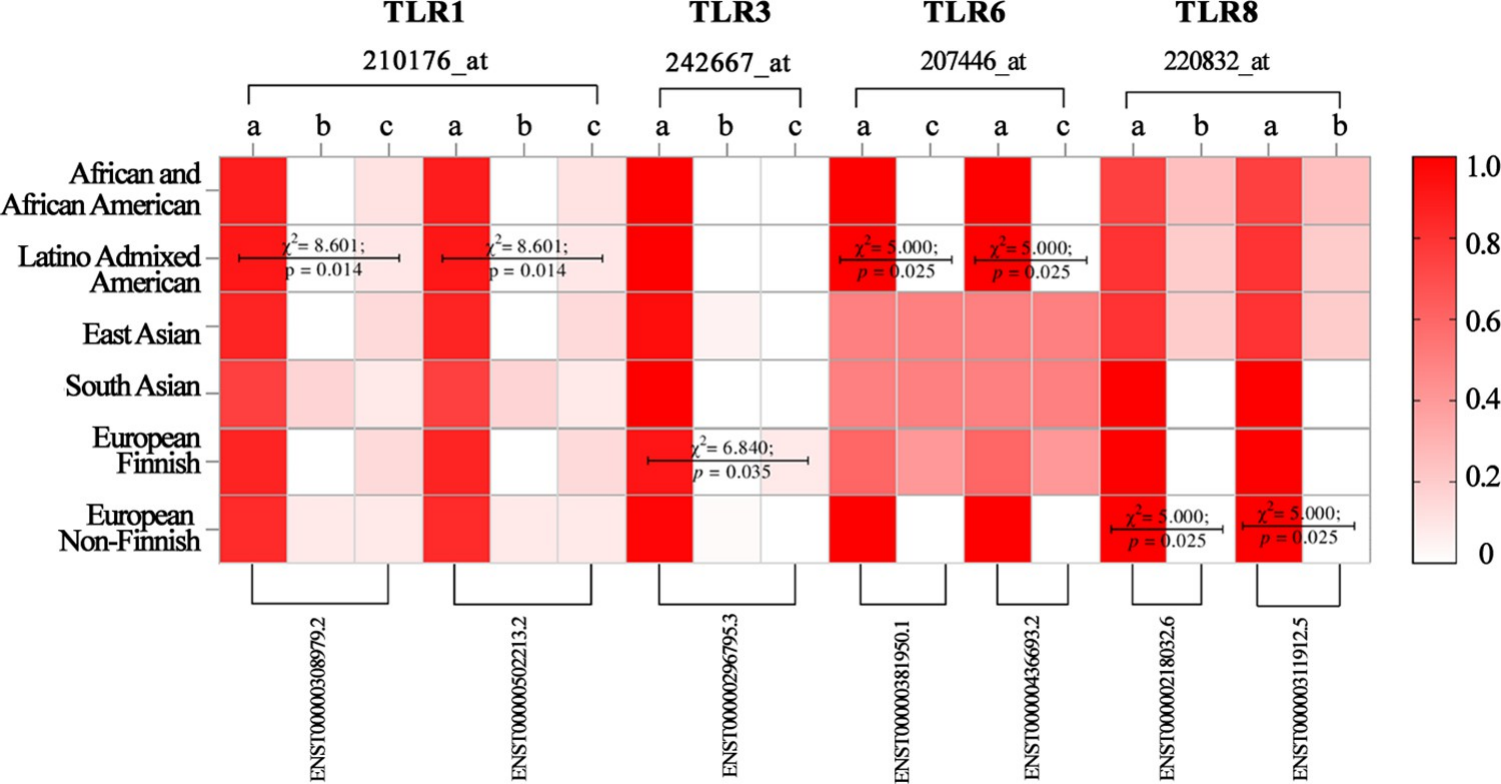
Clinical aspect of the variants observed in the transcripts of TLR1, TLR3, TLR6 and TLR8 from the association of clinical significance with ethnicity. benign; b: pathogenic; c: risk factor; χ^2^: Chi-Square test; p: p-value;.

Fig 2 presents the clinical aspect of the variants found in the transcripts of *TLR1*, *TLR3*, *TLR6* and *TLR8* that were significantly associated with ethnicity.

The ethnicities “Latino Admixed American”, “European Finish” and “European Non-Finnish” presented differences in clinical significance.

In the “Latino Admixed American” population, *TLR1* (Id:210176_at [ENST00000308979.2; ENST00000502213.2]) and *TLR6* (Id:207446_at) [ENST00000381950.1; ENST00000436693.2]) showed a high prevalence of benign variants (Z_crit_ = 2,2).

In addition, *TLR1* had a lower prevalence of pathogenic variants (Z_crit_ = ±2,9), while *TLR6* had a lower prevalence of risk factor variants (Z_crit_ = ±2,2). In the “European Finnish” ethnicity, *TLR3* (Id:242667_at [ENST00000296795.3]) showed a greater prevalence of risk factor variants (Z_crit_ = 2,5). While in the “European Non-Finnish” ethnicity, *TLR8* (Id: 220832 [ENST00000218032.6, ENST00000311912.5) showed a greater prevalence of mutations in the benign categories than in the pathogenic category (Z_crit_ = ±2,2).

### 4. Variants found in transcripts of exomes from different populations

**Fig 3:**
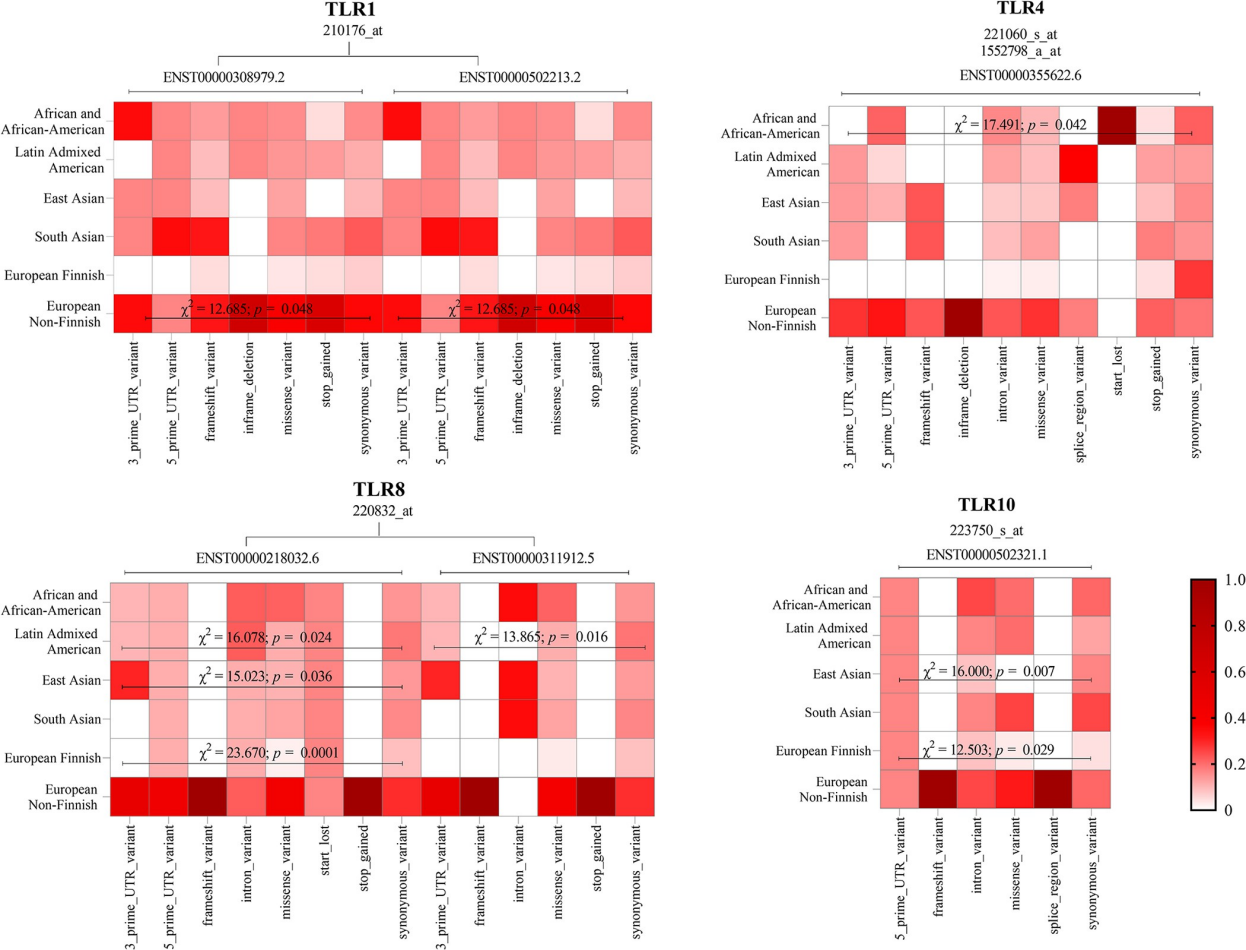
Frequency of variants screened in TLR1, TLR4, TLR8 and TLR10 transcripts from the association of variants with ethnicity. χ^2^: chi-square test; *p*: p value.

The transcript levels of *TLR2*, *TLR3*, *TLR6* and *TLR7* did not significantly differ among the populations (p > 0.05) when the associations between the type of variant and ethnicity were analyzed. Fig 3 shows the differences in the expression of TLRs in the exomes when analyzing the associations between TLRs, which were significant (χ^2^ e Z_crit_).

In *TLR1* [ENST00000308979.2; ENST00000502213.2], there was a difference between the type of variant in the “European Non-Finnish” population, but it was not possible to evaluate the posttest. However, “*5_prime_UTR*” presented a lower frequency than the other types of variants.

There was significant variation in *TLR4* [ENST00000355622.6] in the “African and African American” populations, but this difference was not detected in the posttest. However, “*start_lost*” had a greater prevalence than the others.

Regarding *TLR8*, for ENST00000218032.6, the following changes were observed: “Latino Admixed American”, there was a greater prevalence of variants of the “*star_lost*” and “*synonymous*” types than of the “*missense*” type; “East Asian”, there was a greater frequency of “*3_prime_UTR*” than “*missense*” and “*start_lost*”; “European Finnish”, there was a lower frequency of “*missense*” that “*start_lost*” and “*synonymous*”. For the transcript ENST0000311912.5, there was a greater frequency of “*missense*” and “*synonymous*” variants than of the other variants in the “Latino Admixed American” ethnicity.

Regarding *TLR10* [ENST00000502321.1], it was observed that the “European Finnish” population presented a greater frequency of “*5_prime_UTR*” than the other groups and that in the “East Asian” population, there was a greater frequency of “*5_prime_UTR*” and “*synonymous*” than “*missense*”.

### 5. Relationship between variant type and pathogenetic clinical significance

Subsequently, the investigation of the pathogenic clinical significance of the variants in different populations was carried out on the transcripts that were significant in relation to the previous analysis of the type of variant and ethnicity. Thus, it was found that only the transcripts ENST00000308979.2, ENST00000502213.2, ENST00000218032.6 and ENST00000311912.5 presented events of pathogenic clinical significance and that the mutations from the variant were of the “*missense*” type with alteration of the *A*>*G* alleles on *chromosomes 4* and *X* in the “European Non-Finnish” and “Latino admixed American”, as shown in Table 3.

**Table 3:** Characteristics of the transcripts found to be pathogenic by ethnicity, demonstrating the variation found in the allele.

TLR	Transcript	Variant	Population affected	Chromosome	Position	Allele Count	Allele Number	HGVS Consequence	Protein Consequence	Transcript Consequence	Allele Reference	Allele Alternative
*TLR1*	ENST00000308979.2	*Missense*	European Non-Finnish	*4*	38798417	6	66740	*p*.*Ile679Thr*	*p*.*Ile679Thr*	*c*.*2036T>C*	*A*	*G*
*TLR1*	ENST00000502213.2	*Missense*	European Non-Finnish	*4*	38798417	6	66740	*p*.*Ile679Thr*	*p*.*Ile679Thr*	*c*.*2036T>C*	*A*	*G*
*TLR8*	ENST00000218032.6	*Missense*	Latino Admixed American	*X*	12937211	2	9299	*p*.*Ile18Val*	*p*.*Ile18Val*	*c*.*52A>G*	*A*	*G*
*TLR8*	ENST00000311912.5	*Missense*	Latino Admixed American	*X*	12937211	2	9299	*p*.*Ile36Val*	*p*.*Ile36Val*	*c*.*106A>G*	*A*	*G*

**Fig 4:**
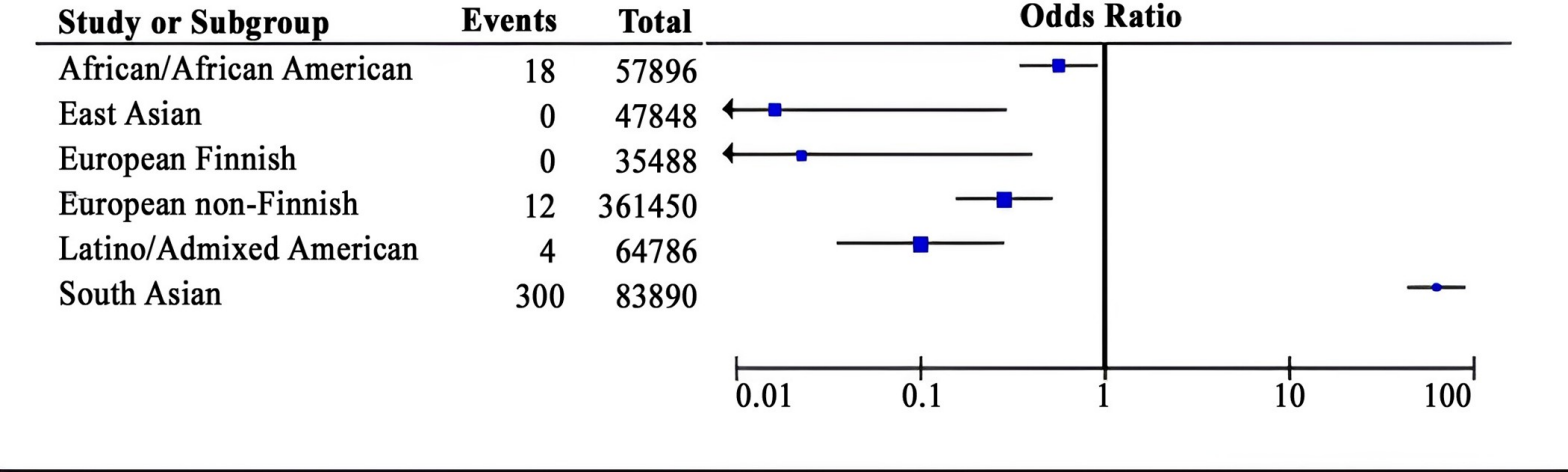
Risk of missense variants with pathogenic characteristics altering allele A>G by ethnicity.

Then, the risk of the “*missense*” variant of pathogenic mutation being found in exomes by ethnicity was calculated based on the proportion of positive alleles for pathogenic characteristics using the odds ratio (OR). Thus, a significantly greater risk was detected in the “South Asian” population (OR = 56.820 [95% CI: 40.206, 80.299]; p < 0.0001), and a lower risk was detected in the “African and African American” (OR = 0.584 [95% CI: 0.3636, 0.9402]; p = 0.031), “European Non-Finnish” (OR = 0.030 [95% CI: 0.0169, 0.0536]; p < 0.0001), “Latino Admixed American” (OR = 0.109 [95% CI: 0.0410, 0.2947]; p < 0.0001), “European Finnish” (OR = 0.025 [95% CI: 0.0016, 0.4164]; p < 0.0001) and “East Asian” (OR = 0.018 [95% CI: 0.0011, 0.3027]; p < 0.0001) (Fig 4). However, the prevalence of *TLR1* was greater (67%).

To determine the quality of the results, we performed a power analysis in G*Power software (latest ver. 3.1.9.7; Heinrich-Heine-Universität Düsseldorf, Düsseldorf, Germany) [22]. One-way ANOVA was used to compare the levels of TLR4 (1552798_a_at) among the control, HPV+ and HPV- groups. The effect size f (1.016) was determined using the data from this TLR. The alpha error was set at 0.05%, and the number of groups = 3. The power of the resulting analysis was 0.997, indicating the scientific value of our results.

## Discussion

Persistent HPV infection is associated with a predisposition to cervical cancer [23, 24]. In contrast, TLRs are groups of molecules capable of responding to and signaling pathways against HPV infection, thus playing a peculiar role between the extremes of protection and the progression of the disease [25]. In this context, considering that cervical cancer is one of the most frequent types of cancer in women [25], research into HPV-induced uterine cervical cancer and the identification of genetic variants in TLRs in population groups can help in screening and identifying ethnicities/races with greater susceptibility to cervical cancer, thus opening up new avenues on the subject and in the field of genetic epidemiology.

In this study, using databases and bioinformatics tools, we analyzed the association of HPV with cervical cancer, the expression pattern of TLRs in HPV+ and HPV- cervical cancer samples, and the existence of TLR variants in exome samples in certain populations. According to the results, we identified a strong association between HPV+ and cervical cancer, showing that the risk of cancer caused by this agent, regardless of age, is high among women [26].

With regard to variations in TLR expression patterns in HPV+ and HPV- samples, we identified alterations in expression patterns in both samples, as well as particularities in cancer type expression. When evaluating the gene expression patterns of TLR transcripts, specifically *TLR1*, *TLR3*, *TLR4*, *TLR6*, *TLR7*, *TLR8* and *TLR10*, in HPV+ and HPV- cervical cancer tissue samples, we observed an increase in *TLR4* expression and a decrease in *TLR1* and *TLR3* expression. According to previous findings, the gene expression of TLRs in cervical tissues is influenced by different types of HPVs, which are able to modulate the host immune response and lead to polymorphisms in TLR genes [27–29]. In addition, the tumor microenvironment, characterized by immunosuppression, also affects the expression of TLRs, impairing the immune response to cervical cancer [30].

Specifically, for *TLR4*, the increase in expression in HPV+ and HPV- samples, particularly with a greater fold change in *TLR4*, regardless of HPV infection, may indicate a response to pathogenicity and the tumor microenvironment, with possible repercussions on the protein and/or processing of the transcript. Alterations in the pattern of *TLR4* expression are associated with the occurrence, development and progression of cervical cancer and are related to HPV infections [10]. However, in this research, we observed that the response also occurs against HPV-. Therefore, this gene should be considered a target for therapeutic tools.

On the other hand, for *TLR1* and *TLR3*, reduced expression was observed in the HPV+ and HPV- samples; however, there was a lower log fold change in the expression in the HPV+ samples. According to a previous study [31], a reduction in *TLR1* expression was detected in the malignant epithelium, and an increase in *TLR3* expression was detected in the premalignant epithelium of the HPV+ cervical cancer tumor microenvironment [31]. Considering that the expression of *TLR1* signals the plasma membrane to the extracellular environment and that *TLR3* is intracellular [32], variations in expression patterns may be related to the microenvironment and tumor development [31]. In our study, the reduction in TLR1 expression in the samples (HPV+ and HPV-) indicates a possible relationship with the stage of tumor development rather than with specificity to the type of cervical cancer.

Particularly in HPV-infected cells, *TLR1* and *TLR3* are negatively regulated compared to those in controls, while *TLR4* and *TLR6* are positively expressed. These results may be related to an attempt by infected cells to initiate immune responses against HPV infection [33], since these markers participate in the response to the activation of proinflammatory cytokines, antiviral responses via interferon regulatory factors and the activation of intracellular signaling pathways to stimulate inflammatory responses [30, 34, 35]. Thus, although *TLR4* is considered an important marker of HPV+ cervical cancer [10], alterations in the levels of *TLR1*, *TLR3* and *TLR6* [33] observed in HPV+ samples may be the target of investigations related to the tumor inflammatory process.

On the other hand, particularities in the expression of TLRs are observed in HPV+ and HPV- samples. Compared with those of the controls, the expression of *TLR7*, *TLR8* and *TLR10* in the HPV+ group was negatively regulated. These results show that HPV+ cells have lower expression of *TLR7*, *TLR8* and *TLR10*. Our results for *TLR7* are similar to those of [27], who assessed the expression levels of genes involved in innate immune responses against HPV and reported a reduction in *TLR7* levels. On the other hand, most studies suggest that the expression of the *TLR7* gene is maintained or increased in HPV-infected mucosa [36, 37]. However, in relation to *TLR8*, previous studies [38–40] contradict our findings, showing increased expression in cervical cancer samples. These genes (*TLR7* and *TLR8*) have been the focus of studies [41–43] because of their immunosuppressive and antitumor functions and can be used as diagnostic biomarkers, indicators of progression and prognosis and immunotherapeutic targets for oncogenesis [43]. The literature reveals that the main functions of *TLR10* are its anti-inflammatory effects and suppressive effects on other tissues [44]. The expression of these markers, specifically in HPV+ cervical cancer, may help in screening and better understanding the mechanisms underlying the development of this type of cancer.

With regard to the analysis to identify clinical variants in TLR exomes with differential expression identified in HPV+ and HPV- samples, we detected variants in certain populations. In the “Latino Admixed American” population, there was a high prevalence of benign variants in *TLR1* (ENST00000308979.2 [Protein coding]; ENST00000502213.2 [Protein coding]) and TLR6 (ENST00000381950.1 [Protein coding]; ENST00000436693.2 [protein coding]). The identification of benign variants in these transcripts may suggest that they are unlikely to cause any disease [45] and could be a protective factor in this population. The functional significance of the identified variant and its contribution to the pathogenesis of cervical cancer have yet to be elucidated.

In contrast, for the “European Finnish” population in relation to *TLR3* (ENST00000296795.3 [Protein coding]), an increase in risk factor variants was observed. Among the pathogenic variants, special attention has been given to risk factor variants [46], as these variants indicate the presence of risk alleles or increased allele frequencies in this specific population, which may be associated with the chance of pathogenicity [46]. Several studies [47–49] have demonstrated the role of *TLR3* in the pathophysiology of cervical cancer, as it plays a critical role in detecting viral infections, such as HPV, and its activation may contribute to a deregulated immune response [47].

On the other hand, in “European Non-Finnish”, a significant number of variants categorized as benign for *TLR8* (ENST00000218032.6 [protein_coding]; ENST00000311912.5 [protein_coding]) may indicate that benign variants are not considered a sufficient cause related to the pathology [50]. Although *TLR8* is a marker related to the activation of innate immunity while inhibiting the regulatory effect of T cells in other gynecological cancers [47], in cervical cancer, it has been observed in other studies that this receptor influences the antitumor immune response and may be an interesting therapeutic target in cervical cancer [37].

Regarding the clinical significance of the [ENST00000218032.6] and [ENST00000311912.5] *TLR8* transcripts, we found a greater prevalence of variants with benign consequences. Although low- to high-impact mutations, such as “*start_lost*”, “*synonymous*” and “*missense*”, have been reported in the “Latino Admixed American” and “European Finnish” ethnic groups, it is possible that they do not affect the protein [51] and therefore do not present a potential risk of developing diseases in these populations. Regarding the prevalence of the “*3_prime_UTR*” in “East Asian” individuals, it is possible that there may or may not be a deregulation at the end of the binding site by the modifying characteristic of this variant [52]; however, this deregulation does not lead to changes in protein expression patterns [53] and therefore has a low potential for developing diseases in these individuals.

In view of the findings on the identification of variants with clinical relevance in exome samples by population, it is noteworthy that in addition to the HPV+ risk factor for the development of cervical cancer [48], the existence of variants in certain TLRs can generate a possible failure in the expression and/or function of TLRs and, consequently, contribute to greater or lesser susceptibility to the onset, development and progression of cervical cancer [37] in specific populations.

On the other hand, when we analyzed the relationship between the type of variant and the clinical pathogenicity of *TLR1* (ENST00000308979.2 [Protein coding]; ENST00000502213.2 [Protein coding]) and *TLR8* (ENST00000218032.6 [protein_coding]; ENST00000311912.5 [protein_coding]) by population, a *missense* variant with an A>G change was identified to be of clinical significance in the “European Non-Finish” and “Latino Admixed American” populations. In the *missense* variant, the substitution of a nucleotide results in an alteration of the amino acid, causing various effects, such as a reduction in the production and/or alteration in the function of the protein [54]. In addition, considering that TLR8 is encoded on the *X chromosome* and expressed and escapes inactivation on the *X chromosome* [55], the presence of variants in the alleles could have severe implications for the immune response related to the tumor microenvironment, thus increasing the vulnerability of certain populations to the onset and progression of the disease [56, 57].

The presence of a *missense* variant with pathogenicity in *TLR1* was also shown to be a high-risk factor in “South Asian” populations, as opposed to the “African and African American”, “European Non-Finish”, “Latino Admixed American”, “European Finish” and “East Asian” populations. These findings may be related to the susceptibility of the populations to this type of variation [58], since this type of mutation causes an alteration in the genetic code of the DNA within the region that codes for a protein, leading to a modification in the corresponding amino acid and, consequently, in the sequence of codons, affecting the structure, function and quantity of the resulting protein [59]. This influences the functionality of TLRs and potentially affects the balance between pro- and anti-inflammatory responses, as well as increasing the risk of chronic infections and cancer development [60, 61].

Furthermore, when we evaluated the types of transcript variants in exomes by population group, we found that “*missense*”, “*start_lost*”, “*synonymous*”, “*3_prime_UTR*” and “*5_prime_UTR*” variants were more frequent in the different populations. Individuals of “African and African American” origin have a greater prevalence of “start_lost” type variants in the transcript (ENST00000355622.6 [protein coding]) belonging to *TLR4*. This type of variant has an impact on the protein chain, which can result in loss of function of the protein due to alteration of the start codon [50], resulting in deregulation of the *TLR4* response to the presence of HPV. This observation could pave the way for further studies to understand how these specific genetic variants affect *TLR4* function and its association with diseases, including cervical cancer, in populations of African origin.

In the “Latino Admixed American” population, we observed that in addition to “*start_lost*” variants in the ENST00000218032.6[protein_coding] transcript, “*synonymous*” and “*missense*” variants were also found in the *TLR8* transcripts (ENST00000218032.6 [protein_coding]; ENST00000311912.5 [protein_coding]).According to the Ensembl platform, the “*synonymous*” variants do not cause changes in the amino acid sequence, and most are assumed to be harmless or unlikely to alter the behavior of the proteins [50]. On the other hand, “*missense*” variants alter one or more bases and are considered variants that can alter the effectiveness of the protein, which may or may not affect protein structure and function [50, 62].

In people of East Asian origin, in the transcripts of *TLR8* (ENST00000218032.6 [protein_coding]) and *TLR10* (ENST00000502321.1[protein coding]), we found “*missense*”, “*start_lost*” and “*synonymous*” variants, which are coding variations that cause changes in the amino acid sequence. In addition, we found the “*3_prime_UTR*” and “*5_prime_UTR*” variants, which are noncoding variations or variations that affect noncoding genes [50]. For the “*3_prime_UTR*”, it is possible that there may or may not be a deregulation at the end of the binding site by the modifying characteristic of this variant [52]; however, this deregulation does not lead to changes in protein expression patterns [53], and therefore has a low potential for developing diseases in these individuals. Although the “*5_prime_UTR*” does not encode a protein directly, it is known to play critical roles in controlling gene expression and regulating protein synthesis. Recent studies highlight the importance of alterations in this variant, which are often associated with the process of oncogenesis [10]. Predictions about the impact of these variants are difficult [50], and there is still much to be studied about their role in the development and progression of cervical cancer.

In “European Finnish”, it was observed that the variants were present in *TLR8* (ENST00000218032.6 [protein_coding]) and *TLR10* (ENST00000502321.1[Protein coding]), however, it was possible to verify a lower prevalence of “*start_lost*”, “*missense*” and “*synonymous*” in *TLR8* and a higher prevalence of “*5_prime_UTR*” in *TLR10* (ENST00000502321.1[Protein coding]). In the European population of non-Finnish origin, we found different variants present in *TLR1*. These findings suggest that these variants may generate polymorphisms in the *TLR1*, *TLR8* and *TLR10* genes in European population groups. It is therefore necessary to emphasize the importance of research into variant screening, as these data will enable new avenues to contribute to the development of resources for cervical cancer detection, diagnosis and therapy.

It is important to recognize that this study has certain limitations, as it uses secondary databases containing the expression levels of transcripts, which are restricted to a certain population, and it is not possible to establish a direct relationship with the existence of TLR variants based on the histological stage of cancer in response to HPV in different population groups. It is suggested that other studies could examine and verify a possible relationship between the expression and existence of TLR variants in samples of histologically differentiated neoplasms. Furthermore, in the analysis to identify genetic variants, it was not possible to obtain sociodemographic data on the individuals, which would allow for greater detail on the epidemiological profile at an individual/population level. Thus, in view of the investigation of the gene expression of TLRs in HPV+ and HPV- patients and of TLR variants in exome samples by population, we believe that despite these limitations, this study contributes to elucidating new research in the area of genetic epidemiology, particularly in cases of cervical cancer. Future studies with large samples could track gene expression and the presence of TLR variants in exome samples in individuals with cervical cancer, with the aim of profiling populations with greater susceptibility to the disease, as well as directing new approaches and inserting detection methods to be considered in possible cervical cancer prevention programs.

## Conclusion

It has been noted that cancer and HPV positivity are associated. With respect to TLRs, *TLR1* and *TLR3* exhibit decreased expression independent of HPV and are more altered in HPV-positive patients. In addition, *TLR1* occurs less frequently as “*5_prime_UTR*” in exomes of the “European Non-Finnish” population, with most cases occurring in the “Latino Admixed American” population of benign significance and a higher prevalence of pathogenic cases with “*missense*” mutation occurring in the “South Asian” population. *TLR3* also represents a greater risk factor in the “European Finnish” population. *TLR4* is more highly expressed in HPV-independent tumor tissue, and the number of “star_lost” variants is greater in “African and African-American populations. *TLR6* showed greater expression in HPV-positive individuals, but there was a greater amount of benign development in the “Latino Admixed American” population. *TLR7*, *TLR8*, and *TLR10* were expressed at low levels in HPV-positive tissue. *TLR8* shows a prevalence of benign variants, and in exomes, the mutations “*3_prime_UTR*” (East Asian), “*start_lost*” (Latino Admixed American and European Finnish), “synonymous” (Latino Admixed American and European Finnish), and “*missense*” (Latino Admixed American) are found. *TLR10* has more “*5_prime_UTR*” (European Finnish and East Asian) and “*synonymous*” (East Asian) mutations.

## Institutional review board statement

The present study was exempt from ethical review and approval because the dataset used includes only aggregate data and does not reveal personal information of individuals.

## Supporting information

S1 Appendix Data of research. (XLSX)
